# Preoperative biliary drainage for periampullary tumors causing obstructive jaundice; *DR*ainage vs. (direct) *OP*eration (DROP-trial)

**DOI:** 10.1186/1471-2482-7-3

**Published:** 2007-03-12

**Authors:** Niels A van der Gaag, Steve MM de Castro, Erik AJ Rauws, Marco J Bruno, Casper HJ van Eijck, Ernst J Kuipers, Josephus JGM Gerritsen, Jan-Paul Rutten, Jan Willem Greve, Erik J Hesselink, Jean HG Klinkenbijl, Inne HM Borel Rinkes, Djamila Boerma, Bert A Bonsing, Cees J van Laarhoven, Frank JGM Kubben, Erwin van der Harst, Meindert N Sosef, Koop Bosscha, Ignace HJT de Hingh, Laurens Th de Wit, Otto M van Delden, Olivier RC Busch, Thomas M van Gulik, Patrick MM Bossuyt, Dirk J Gouma

**Affiliations:** 1 Department of Surgery, Academic Medical Center Amsterdam, the Netherlands; 2 Department of Gastroenterology, Amsterdam, the Netherlands; 3 Department of Surgery, Erasmus Medical Center, Rotterdam, the Netherlands; 4 Department of Gastroenterology, Erasmus Medical Center, Rotterdam, the Netherlands; 5 Department of Surgery, Medical Spectrum Twente, Enschede, the Netherlands; 6 Department of Surgery, University Hospital Maastricht, Maastricht, the Netherlands; 7 Department of Surgery, Gelre Hospital, Apeldoorn, the Netherlands; 8 Department of Surgery, Rijnstate Hospital, Arnhem, the Netherlands; 9 Department of Surgery, University Medical Center Utrecht, the Netherlands; 10 Department of Surgery, St Antonius Hospital, Nieuwegein, the Netherlands; 11 Department of Surgery, Leiden University Medical Center, the Netherlands; 12 Department of Surgery, St Elisabeth Hospital, Tilburg, the Netherlands; 13 Department of Gastroenterology, Medical Center Rijnmond Zuid, Rotterdam, the Netherlands; 14 Department of Surgery, Medical Center Rijnmond Zuid, Rotterdam, the Netherlands; 15 Department of Surgery, Atrium Medical Center, Heerlen, the Netherlands; 16 Department of Surgery, Jeroen Bosch Hospital, 's Hertogenbosch, the Netherlands; 17 Department of Surgery, Catharina Hospital, Eindhoven, the Netherlands; 18 Department of Surgery, Onze Lieve Vrouwe Gasthuis, Amsterdam, the Netherlands; 19 Department of Radiology, Academic Medical Center Amsterdam, The Netherlands; 20 Department of clinical epidemiology and biostatistics, Academic Medical Center Amsterdam, the Netherlands

## Abstract

**Background:**

Surgery in patients with obstructive jaundice caused by a periampullary (pancreas, papilla, distal bile duct) tumor is associated with a higher risk of postoperative complications than in non-jaundiced patients. Preoperative biliary drainage was introduced in an attempt to improve the general condition and thus reduce postoperative morbidity and mortality. Early studies showed a reduction in morbidity. However, more recently the focus has shifted towards the negative effects of drainage, such as an increase of infectious complications. Whether biliary drainage should always be performed in jaundiced patients remains controversial. The randomized controlled multicenter DROP-trial (DRainage vs. Operation) was conceived to compare the outcome of a 'preoperative biliary drainage strategy' (standard strategy) with that of an 'early-surgery' strategy, with respect to the incidence of severe complications (primary-outcome measure), hospital stay, number of invasive diagnostic tests, costs, and quality of life.

**Methods/design:**

Patients with obstructive jaundice due to a periampullary tumor, eligible for exploration after staging with CT scan, and scheduled to undergo a "curative" resection, will be randomized to either "early surgical treatment" (within one week) or "preoperative biliary drainage" (for 4 weeks) and subsequent surgical treatment (standard treatment). Primary outcome measure is the percentage of severe complications up to 90 days after surgery. The sample size calculation is based on the equivalence design for the primary outcome measure. If equivalence is found, the comparison of the secondary outcomes will be essential in selecting the preferred strategy. Based on a 40% complication rate for early surgical treatment and 48% for preoperative drainage, equivalence is taken to be demonstrated if the percentage of severe complications with early surgical treatment is not more than 10% higher compared to standard treatment: preoperative biliary drainage. Accounting for a 10% dropout, 105 patients are needed in each arm resulting in a study population of 210 (alpha = 0.95, beta = 0.8).

**Discussion:**

The DROP-trial is a randomized controlled multicenter trial that will provide evidence whether or not preoperative biliary drainage is to be performed in patients with obstructive jaundice due to a periampullary tumor.

## Background

Patients with obstructive jaundice caused by a tumor in the pancreatic head area (pancreas, distal bile duct, papilla of Vater), without radiological evidence of irresectability, will undergo an exploration with the intention of resection of the tumor, being the only option for cure [1-4]. If a resection is not possible due to locoregional irresectability or distant metastases, a biliary and gastric bypass procedure is performed [5-8]. Surgery in jaundiced patients with a tumor in the pancreatic head area is associated with a higher risk of postoperative complications compared with surgery in non jaundiced patients [9-11]. These complications primarily consist of septic complications (cholangitis, abscesses, and leakage), haemorrhage, impaired wound healing and renal disorders. The increased risk of surgery in jaundiced patients was recognized already in 1935 by Allen O. Whipple, who proposed a two staged procedure for surgery in deeply jaundiced patients. The first stage consisted of a drainage procedure by a cholecystogastrostomy, to decompress the biliary tract in order to restore normal liver function, followed four weeks later by radical resection of the tumor [12]. Numerous experimental and clinical studies have been performed since, investigating the cause and directed at prevention of complications after surgery in patients with obstructive jaundice.

While the postoperative mortality rate after pancreatoduodenectomy has been reduced from around 20% to 1–5% in experienced centres, the morbidity rate has remained virtually unchanged, ranging from 40 to 60% [5,6,8,9]. Many different etiologic factors for development of complications have been characterized: presence of toxic substances as bilirubin and bile salts, impaired nutritional status, effects of endotoxins, bacterial translocation, modulation of the inflammatory cascade with cytokine release, reduction of cellular immunity and nutritional status [13-21]. For these etiologic factors different interventions have been undertaken in the past decades, attempting to lower the risk of complications. The current project addresses the issue of preoperative biliary drainage (PBD).

Early studies on external PBD could not demonstrate a reduction in complication rate in humans because this procedure, although relieving the biliary obstruction, does not restore the bile flow to the gut lumen [22]. Internal drainage has been shown in multiple experimental models to improve liver function and nutritional status, to reduce systemic endotoxemia and cytokine release, and subsequently to improve immune response [13-15,19-21]. Finally the mortality was significantly reduced in these animal models[16]. The first non-randomized studies on internal PBD in jaundiced patients reported a reduced mortality and morbidity [22]. However, clinical studies and small randomized trials could not confirm the positive effect of PBD on surgical outcome [23-27]. Some studies even reported a deleterious effect, partly due to complications associated with the drainage procedure [28-32]. Despite these results, PBD is generally accepted in The Netherlands. In previous studies we found that around 90% of patients with obstructive jaundice currently undergo preoperative drainage in The Netherlands [28,29]. The dominance of drainage can be attributed in part to the familiarity of endoscopic retrograde cholangiopancreatography (ERCP), which has been used in the past as the first diagnostic procedure for obstructive jaundice. The time needed for extensive diagnostic workup, including diagnostic laparoscopy (DL), is a logistic explanation for the assumed benefit of PBD. However, the value of DL for periampullary tumors is disputed, especially in the light of improvement of other non-invasive diagnostic procedures [1-4,33,34]. Other important factors that influence the decision to opt for PBD are (local) referral patterns and waiting lists. Current state-of-the-art radiological diagnostic and staging procedures for suspected periampullary tumors require only a minimum of time [1]. These non-invasive radiological procedures have the same diagnostic accuracy as ERCP, an invasive diagnostic procedure, and, moreover, offer the advantage of assessing local tumor extension as well as distant metastases [1-3]. Therefore, ERCP with subsequent drainage as part of a routine diagnostic workup is outdated.

ERCP and endoscopic sphincterotomy with insertion of biliary and pancreatic stents is a difficult gastrointestinal endoscopic procedure. Complications, such as haemorrhage, pancreatitis, perforation of the duodenal wall, cholangitis and stent occlusion cannot always be avoided and occur in approximately 10 percent of procedures [35,36]. Mortality as a consequence of the procedure is reported in 0.5%–1% of the cases [36]. The negative side-effects of PBD, such as an increase of infectious complications after surgery, has been the focus of attention in more recent studies [30-32]. It was concluded that the potential advantages of preoperative drainage fail to outweigh the negative effects [20,23,24,28-32].

In the light of the ongoing controversy of PBD, a meta-analysis of randomized clinical trials and comparative studies was carried out [37]. The aim of this study was to evaluate the efficacy of drainage in jaundiced patients compared with patients that underwent direct surgical treatment. No difference in mortality could be detected between both strategies, but overall complication rate in patients that underwent PBD was significantly higher compared with direct surgical treatment, 57.3% and 41.9% respectively (level I evidence). The mean overall hospital stay was increased by two weeks in patients that underwent biliary drainage. Unfortunately, most of the studies have methodological flaws (e.g. differences in drainage procedures, duration of drainage, internal vs. external drainage, surgical procedures, small sample size) and do not provide unequivocal treatment recommendations. Therefore, a prospective randomized trial addressing the effects of PBD is indicated. Especially, for the potential consequences of future treatment might be considerable; a shorter workup period, less invasive diagnostic procedures (ERCP) and a shorter time interval to surgery. The study focuses on complication rate (40–60%), the primary endpoint for most past studies.

## Methods/Design

### Study objectives

The proposed project involves a randomized multicenter trial to compare the outcome of "a preoperative biliary drainage strategy" (standard strategy) with that of an "early surgery" strategy, with respect to the incidence of severe complications (primary outcome measure), hospital stay, number of invasive diagnostic tests and costs.

### Study design

**Figure 1:**
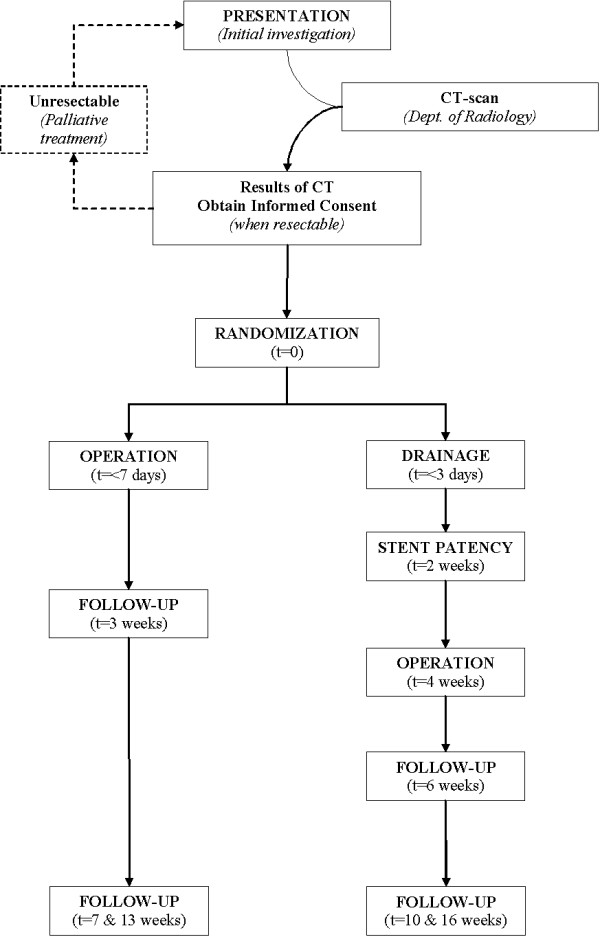
DROP-study flowchart.

The DROP-trial is a randomized multicenter trial with four academic and ten regional hospitals participating in the project. Patients with obstructive jaundice (bilirubin level 40–250 μmol/l) due to a pancreatic/periampullary tumor, who are candidate for exploration after staging with CT scan, and scheduled to undergo a "curative" resection, will be randomized to either "early surgical treatment" (within one week) or "preoperative biliary drainage" (for four weeks) and subsequent surgical treatment (standard treatment) (figure 1). Randomization is centralized in the Academic Medical Center, Amsterdam, by means of a computer generated allocation with stratification for center.

### Study population

The study population consists of patients with the clinical diagnosis of obstructive jaundice due to a pancreatic head or periampullary tumor. Inclusion criteria are; a serum bilirubin level of > 40 μmol/l and < 250 μmol/l at randomization, CT without evidence of distant metastases or local tumor ingrowth into portal or mesenteric vessels (as defined by the tumor surrounding the vessel for at least 180 degrees of the circumference), referred for surgical treatment to one of the participating centres, time between CT and randomization ≤ 4 days, informed consent. Exclusion criteria are; age > 85 years or severe co-morbidity (Karnofsky <50%) and other contraindications for major surgery, cholangitis/infection, previous ERCP and stenting or percutaneous biliary drainage, previous chemotherapy for this malignancy, severe gastric outlet obstruction (stenosis duodenum due to tumor ingrowth) defined as vomiting, nausea and/or oral intake less than one l/day.

### Primary outcome parameter

Primary outcome parameter are severe complications and the study is designed for detecting equivalence in the occurrence of these events during treatment within 120 days after randomization. This will include at least 90 days follow-up after surgery, a period selected as adequate, while > 95% of complications will occur within 30 days after surgery. A longer observation period up to one year will potentially include effects of progressive or recurrent disease.

A severe complication is defined as any complication, related to the drainage procedure and surgical treatment, leading to an additional invasive intervention or relaparotomy with subsequent prolonged hospital stay or death, or readmission for disease related morbidity within 120 days after randomization (table 1). The definition of all these complications, as well as their management, have been extensively evaluated in previous studies in our institution during the past 10 years according to generally accepted criteria [5,28,29,35,36,38,39].

**Table 1:** Severe ERCP- and surgery-related (postoperative) complications [5,28,29,35,36,38,39,43,44].

	***COMPLICATION***	***CRITERIA***
***ERCP***		
	- Acute pancreatitis	Abdominal pain and a serum concentration of pancreatic enzymes (amylase or lipase) two or more times the upper limit of normal, that required more than one night of hospitalisation
	- Cholangitis	Elevation in the temperature to more than 38°C, thought to have a biliary cause, without concomitant evidence of acute cholecystitis
	- Acute cholecystitis	No suggestive clinical or radiographic signs of acute cholecystitis before the procedure and if emergency cholecystectomy is subsequently required
	- Perforation	Retroperitoneal or bowel-wall perforation documented by any radiographic technique
	- Haemorrhage	Clinical evidence of bleeding (melena or hematemesis) with an associated decrease of at least 2 g per decilitre in the haemoglobin concentration, or the need for a blood transfusion
	- Stent Occlusion	Recurring obstructive jaundice with necessary stent replacement
***SURGERY***		
	- Pancreatojejunostomy leakage	Drain output of any measurable volume of fluid on or after postoperative day 3 with an amylase content greater than 3 times the serum amylase activity, graded according to clinical course (ISGPS grade A, B, C)
	- Postpancreatectomy haemorrhage	Bleeding after the index operation requiring ≥ 4 units of packed cells and/or leading to relaparotomy/intervention
	- Delayed gastric emptying	Gastric stasis requiring nasogastric intubation for 10 days or more, or the inability to tolerate a regular (solid) diet on or before the fourteenth postoperative day
	- Biliary leakage	Bilirubin in abdominal drain or dehiscence found at laparotomy
	- Sepsis	Presence of two or more of the following: fever or hypothermia, leucocytosis or leucopenia, tachycardia, and tachypnea or a supernormal minute ventilation
	- Intra-abdominal abscess formation	Intra-abdominal fluid collection with positive cultures identified by ultrasonography or computed tomography, associated with persistent fever and elevations of white blood cells
	- Wound infection	Requiring intervention with subsequent prolonged hospital stay, otherwise considered as minor complication
	- Burst abdomen	
	- Any relaparotomy for other reasons	
	- Pneumonia	

To exclude bias in determining events of the primary endpoint, a blinded adjunction committee will review all events and evaluate whether events account as severe complications (with intervention, relaparotomy) needing prolonged hospital stay, or even a readmission.

### Secondary outcome parameters

Secondary endpoints are hospital stay, the number of (invasive) diagnostic procedures, medical and non medical costs, and quality of life. Quality of life will be measured for patients treated at the trial centre, at fixed intervals, by two validated questionnaires; the European Organisation for Research and Treatment of Cancer (EORTC) Quality of Life Questionnaire C-30 (QLQ-C30) and its Pancreatic Cancer Module (QLQ-PAN26) [40].

### Participating centres

Fourteen Dutch hospitals of the DROP-Trial group, including four academic centres and ten non-academic centres, are currently participating in this trial.

### Ethics

This study is conducted in accordance with the principles of the Declaration of Helsinki and 'good clinical practice' guidelines. The independent medical ethics committees of the participating hospitals have approved the study protocol. Prior to randomization, written informed consent will be obtained from all patients.

### Study outline

Patients may be included if CT scan has demonstrated a lesion in the pancreatic head area without metastases and/or local tumor ingrowth. Local physicians/gastroenterologists can refer patients for the study by contacting the primary trial centre or one of the participating centres (see addendum 1). Written informed consent will be obtained at the outpatient department according to the Guidelines of Clinical Research in Humans, when the patient meets the in- and exclusion criteria. Randomization is performed instantly by the trial centre.

After randomization, patients will be scheduled, either for surgical treatment within one week, or PBD with subsequent surgical treatment after four weeks. PBD is preferably performed by local gastroenterologists or in the referral centre according to local policy. The participating centres accepted to arrange extra operating room capacity to guarantee early surgery to be carried out when indicated.

#### ERCP + PBD

- ERCP with stent placement will be performed by an experienced endoscopist, by insertion of a single (plastic) stent

- If ERCP is not successful, the patient is to be referred to a tertiary centre for a second attempt for endoscopic drainage, or a percutaneous drainage will be performed, according to local preference and expertise.

- Biliary drainage is considered adequate if a decrease of > 50% of serum bilirubin level is found after two weeks of drainage; otherwise the stent should be changed.

- After four weeks of PBD, patients will undergo surgery. In case of complications indicating inadequate bile drainage and stent obstruction (cholangitis, stent occlusion) a stent exchange will be performed.

- Other complications, such as bleeding or severe pancreatitis, are treated according to the local generally accepted guidelines and could consequently lead to a delay of surgery [35,36].

- Preoperative nutritional support (e.g. consultation with a dietician) is recommended in patients with a weight loss of more than 15% during the last 3 months.

#### Surgery

- Surgery is planned keeping in mind the maximum estimated bilirubin level (> 40 μmol/l and < 250 μmol/l at randomization) must not exceed 300 μmol/l, 24 hours before surgery (e.g. high bilirubin at randomization requires earlier surgery).

- Vitamin K (10 mg, oral, 1 day preoperatively) is given on indication, cefuroxim (1500 mg, intravenous single shot, 1/2 hour preoperatively) and octreotide or analogues (3 × 100 μg, subcutaneous, 12 hours before surgery and continued for seven days after surgery) as prophylaxis.

- During exploration the standard procedure will be the standard pylorus preserving pancreatoduodenectomy (PPPD), as previously described, with removal of lymph nodes at the right side of the portal vein [33]. If indicated (suspicious ingrowth proximal duodenum/pylorus) a Whipple procedure is to be performed. In case of limited vascular ingrowth, a (wedge) resection of the portal/mesenteric vein might be performed [33,41].

- Reconstruction is performed by pancreaticojejunostomy, a hepaticojejunostomy and gastrojejunostomy [33].

- One silicone drain is placed near the pancreaticojejunostomy and/or one near the hepaticojejunostomy; T-drains will not be used.

- A feeding jejunostomy is not to be used as standard treatment

- If resection is not performed due to metastases or local ingrowth, biopsies have to be taken for a histological diagnosis.

Palliative treatment consist of a hepaticojejunostomy with gastroenterostomy plus a celiac plexus blockade [7,33,42]. If a hepaticojejunostomy is not possible, a Wallstent is inserted postoperatively by means of ERCP.

### Statistical analysis

#### Intention to treat

The analysis will be performed in accordance with the intention to treat principle.

#### Sample size calculation

The recently performed meta-analysis of randomized clinical trials and comparative studies, addressing the effectiveness of a PBD strategy in jaundiced patients versus a direct surgical treatment, did not show a difference in mortality between the two strategies [37]. The overall complication rates were, summarizing level I and II studies, 42% for the 'early surgery' strategy and 58.1% for strategy with preceding PBD.

The sample size calculation is based on the equivalence design for the primary endpoint, severe complications of treatment. Based on an expected 38% complication rate for early surgical treatment and 48% for preoperative drainage, equivalence is taken to be demonstrated if the percentage of severe complications with early surgical treatment is not more than 10% higher compared to standard treatment with PBD. Accounting for a 10% dropout, 105 patients are needed in each arm resulting in a study population of 210 (alpha = 0.95, beta = 0.8). If equivalence is found, the comparison of the secondary outcomes will be essential in selecting the preferred strategy.

### Data collection and monitoring

All postoperative complications will be monitored during hospital stay and follow-up of 2, 6, 12 weeks after discharge. The questionnaires (QLQ-C30/PAN-26) are filled in by the patient on the day of randomization, after two weeks of drainage when randomized for PBD, and three and six weeks after discharge from admission for surgery. There will be regular contact between the trial coordinator and the participating centres. The trial coordinator will monitor the data of every included patient.

### Data analysis

The study is designed as an equivalence trial with respect to the primary outcome measure and superiority with respect to the secondary outcome measures. The principal analysis consists of an intention-to-treat comparison of the severe complication rate in both treatment strategies. The comparison will be expressed in terms of a relative risk and 95% confidence intervals. The research hypothesis will be evaluated using a two-group large-sample normal approximation test of proportions, with a one-sided 0.05 significance level. In addition, a comparison of the incidence of complications over time will be made using Kaplan-Meier estimates.

Subsequent analyses are directed at the secondary endpoints. The total number of additional procedures, the length of hospital stay, hospital-free survival and total costs will be compared using t-test statistics. Preplanned subgroup analyses include type of surgery (resection versus bypass), severity of jaundice and other risk factors for complications such as age > 70 years, weight loss > 10%, low albumin levels, renal function, low Hb (< 7 mmol/l), tumor pathology (papilla neoplasms, pancreatic head neoplasms and distal bile duct neoplasms).

An independent safety committee, consisting of three specialists (dept of internal medicine, dept of surgery, dept of clinical statistics), will guarantee the safety of the patients. After 105 included patients (50%), a blinded interim-analysis will be performed of all endpoints. Because of the delayed response for determination of the primary endpoint (severe complications), this analysis will be performed 120 days after randomization of the last patient to reach n: 105. The nominal significance (sequential two-sided testing) for the stopping rule is p < 0.01 (α = 0.01) for adverse effects (severe complications principal endpoint of safety analysis). Analysis will be performed for the individual groups and for the principal adverse effects.

### Economic evaluation

If the assumption of equivalence holds, the economic evaluation can be designed as a cost-minimization analysis from a societal perspective. Given equivalence in survival for the two strategies, the time horizon for the evaluation can be limited to 120 days after randomization.

According to guidelines for cost research, the two treatment strategies will be compared from the societal perspective, regarding direct medical and non-medical and indirect non-medical costs. In the economic evaluation direct medical and non-medical costs and indirect costs are related to the primary endpoints, reflecting economic efficiency at 3 months after admission.

We expect early surgery without preoperative biliary drainage to lead to a shorter hospital stay, less invasive diagnostic tests, fewer additional procedures and, hence, lower costs. Nowadays overall mortality after PD is around 2% for control patients (drainage) and therefore an increase or reduction of mortality can only have limited influence on the primary outcome parameter (severe complications and death). As we could not find any data to provide even an indication of economic outcomes of both strategies, the evaluation will be designed as a cost-effectiveness analysis.

To document cumulative three-month total costs for both treatment strategies, we will track the use of resources, using hospital information systems and additional data collection in the case record forms. The tracking of resources will start at randomization (i.e. after preoperative staging has been completed). Unit prices will either be determined based on current guidelines for economic evaluations or, alternatively, if not existent or not applicable, they will be calculated during the study. Out of hospital resource use, as well as data on direct non-medical and indirect costs will not be analyzed. Within the study, differences in treatment will be analyzed to detect potential limitations to the reproducibility of our findings.

## Discussion

The concept of PBD has been developed to reduce the postoperative morbidity and mortality in patients with obstructive jaundice, caused by a suspected pancreatic/periampullary malignancy. However, PBD, both endoscopic or percutaneous, is associated with an increased incidence of postoperative morbidity [mostly infectious complications] and postoperative mortality when performed prior to a pancreatoduodenectomy. Furthermore, the techniques used for PBD harbour their own complications. Therefore, the overall conclusion not to routinely perform preoperative biliary drainage seems evident. Nevertheless, still the majority of these patients undergo preoperative drainage, often preceded by a diagnostic endoscopic retrograde cholangiopancreatography (ERCP). Unfortunately, most of the available literature addressing the efficacy of PBD suffers from methodological flaws (e.g. differences in drainage procedures, duration of drainage, internal vs. external drainage, surgical procedures, small sample size) or is outdated.

Therefore, a prospective randomized trial addressing the effects of PBD, and thus solving the longstanding controversy whether or not PBD should be routinely performed in jaundiced patients prior to a pancreatoduodenectomy, is indicated. Especially, for the potential consequences of future treatment might be considerable; a shorter workup period, less invasive diagnostic procedures (ERCP) and a shorter time interval to surgery.

## Abbreviations

DROP-trial: DRainage vs. (direct) Operation

PBD: preoperative biliary drainage

ERCP: endoscopic retrograde cholangiopancreatography

EORTC QLQ-C30: European Organisation for Research and Treatment of Cancer (EORTC) Quality of Life Questionnaire C-30

EORTC QLQ-PAN26: European Organisation for Research and Treatment of Cancer (EORTC) Quality of Life Questionnaire PANcreas-26

## Competing interests

The author(s) declare that they have no competing interests.

## Authors' contributions

NAvdG and SMMC drafted the manuscript. DJG is the principal investigator of this study and co-authored the writing of the manuscript. PMMB, OMD, CHJE, EJK, JJGMG, JWG, EJH, JHGK, CJHML, LTW, MJB EAJW participated in the design of the study during several meetings and are local investigators at the participating centres. The other authors are local investigators. All authors edited the manuscript and read and approved the final manuscript.

## Pre-publication history

The pre-publication history for this paper can be accessed here:


